# Efficacy of PD-1/PD-L1 inhibitors in patients with advanced gastroesophageal cancer: An updated meta-analysis based on randomized controlled trials

**DOI:** 10.3389/fphar.2022.1009254

**Published:** 2022-10-25

**Authors:** Lihu Gu, Tongmin Huang, Shinan Qiu, Jiaze Hong, Rongrong Fu, Chaoxiong Ni, Senjie Dai, Ping Chen, Ning He

**Affiliations:** ^1^ Department of General Surgery, Ningbo No. 2 Hospital, Ningbo, Zhejiang, China; ^2^ Ningbo Institute of Life and Health Industry, University of Chinese Academy of Sciences, Ningbo, China; ^3^ Key Laboratory of Diagnosis and Treatment of Digestive System Tumors of Zhejiang Province, Ningbo No. 2 Hospital, Ningbo, China; ^4^ The Second Clinical Medical College, Zhejiang Chinese Medical University, Hangzhou, Zhejiang, China; ^5^ Medical School of Ningbo University, Ningbo, Zhejiang, China; ^6^ The First Clinical Medical College, Zhejiang Chinese Medical University, Hangzhou, Zhejiang, China; ^7^ Department of Nephrology, QingChun Hospital of Zhejiang Province, Hangzhou, Zhejiang, China; ^8^ Department of Tumor High-Intensity Focused Ultrasound (HIFU) Therapy, Ningbo No. 2 Hospital, Ningbo, Zhejiang, China

**Keywords:** PD-1/PD-L1 inhibitors, gastroesophageal cancer, survival, clinicopathological features, sex, meta-analysis

## Abstract

**Background:** This study aimed to investigate the clinical efficacy of programmed death-1 receptor and ligand-1 (PD-1/PD-L1) inhibitors in gastroesophageal cancer patients and the relationship between their clinicopathological features and curative treatment effects.

**Methods:** A systematic search was conducted for articles published before April 2022 from online databases (PubMed, EMBASE, Web of Science and the Cochrane Library). The main outcome was overall survival (OS).

**Results:** This meta-analysis comprised 16 studies involving 9,304 participants. The results indicated that compared with chemotherapy, patients treated with PD-1/PD-L1 inhibitors had significantly improved OS (HR = 0.80; *p* < 0.001) but no significant improvement in progression-free survival (PFS) (*p* = 0.185). Subgroup analyses demonstrated that PD-1/PD-L1 inhibitors combined with chemotherapy, esophageal squamous cell carcinoma, male, Asian patients and combined positive score (CPS) ≥1 were significantly associated with better survival outcomes. Further, subgroup analysis of gender revealed that the OS of all subgroups containing male patients was significantly improved compared with chemotherapy, unlike that of female patients. In addition, the line of therapy, Lauren classification, age and eastern cooperative oncology group (ECOG) performance status were not associated with PD-1/PD-L1 inhibitors efficacy.

**Conclusion:** The results indicated that PD-1/PD-L1 inhibitors could prolong the OS of advanced gastroesophageal cancer patients. Clinicopathological features such as therapeutic schedules, tumor types, histological type, gender, geographical region and PD-L1 expression status (CPS) seemed to be associated with survival outcomes.

## Introduction

Gastroesophageal cancer, one of the most lethal malignant tumors with a dismal prognosis, can be anatomically separated into esophageal cancer, gastroesophageal junction cancer and gastric cancer. As per the GLOBOCAN 2020 assessment of cancer incidence and mortality (Sung et al., 2021), it is estimated that there will be approximately more than 540,000 cases of esophageal cancer and 760,000 cases of gastric cancer deaths each year, making esophageal cancer and gastric cancer the sixth and third causes of cancer mortality globally. Due to the indolent course of the disease in its early stage, most gastroesophageal cancer patients have already reached advanced stages by the time of diagnosis and thereby have dismal prognoses (Ajani et al., 2016; Van Cutsem et al., 2016). Consequently, single or combination therapy with chemotherapy, targeted therapy and radiotherapy are the main methods to enhance the survival and quality of life of the patients. Currently, the first-line treatment for advanced gastroesophageal cancer consists of 5-FU/platinum-based chemotherapy and targeted therapy. Nonetheless, chemotherapy, targeted therapy, and radiotherapy have been reported to have limited efficacy, with 5-years survival rates ranging from 15% to 25% (Zou et al., 2016; Bray et al., 2018).

Recently, immune checkpoint inhibitors (ICIs), particularly programmed death-1 receptor and ligand-1 (PD-1/PD-L1) inhibitors, have shown promising prospects in prolonging the survival of advanced cancer. By blocking related immune checkpoint signaling pathways, ICIs can restore the anti-tumor immune responses of immune cells (Sharma and Allison, 2015). The Food and Drug Administration (FDA) has approved the use of numerous PD-1/PD-L1 inhibitors for the clinical treatment of some cancers (Ni et al., 2020). For instance, pembrolizumab in combination with trastuzumab and chemotherapy for first-line treatment of metastatic HER-2 positive gastric cancer, and nivolumab combined with chemotherapy in the first-line setting for the treatment of advanced gastroesophageal cancer (Weadick et al., 2022). However, since the response rates to PD-1/PD-L1 inhibitors remain unsatisfactory in most patients, this has restricted their clinical applications (Yi et al., 2018). Some scholars have turned their attention to exploring markers that could effectively predict the efficacy of PD-1/PD-L1 inhibitors, with the hope of screening latent patients who would effectively respond to PD-1/PD-L1 inhibitors before treatment; thus, improving their treatment outcomes.

Previously, subgroup analyses of markers affecting the efficacy of PD-1/PD-L1 inhibitors in gastroesophageal cancer were performed through two meta-analyses (Formica et al., 2021; Oh et al., 2021), but given the limited number of studies included, inconsistency of research design and significant heterogeneity among the included studies, the credibility of their results and conclusions seemed limited. Hence, based on the latest evidence from randomized controlled trials (RCTs), an updated meta-analysis will be executed to probe the clinical efficacy of PD-1/PD-L1 inhibitors in advanced gastroesophageal cancer and the association between patients' clinicopathological features (therapeutic schedules, sex, age, etc.) and curative treatment effects depression.

## Methods

This meta-analysis was carried out in accordance with the preferred reporting items for Systematic Review and Meta-Analysis (PRISMA) 2015 (Moher et al., 2015), and the prospective protocol was registered on the PROSPERO (CRD42022327617).

### Search strategy

Two authors separately conducted a systematic search of PubMed, EMBASE, Web of Science and the Cochrane Library to identify all potentially RCTs relevant to the efficacy of PD-1/PD-L1 inhibitors in esophageal cancer, gastroesophageal junction cancer and gastric cancer. And the scope of the literature search was restricted to the time span from inception of the databases to April 2022. Random combinations of free-text terms and medical subject headings terms were used to retrieve literature. And search terms included “PD-1” OR “PD-L1” OR “programmed death 1” OR “programmed death ligand 1” OR “nivolumab” OR “BMS 936558” OR “BMS 936559” OR “MDX 1105” OR “pembrolizumab” OR “lambrolizumab” OR “MK 3475” OR “pidilizumab” OR “CT 011” OR “durvalumab” OR “MEDI 4736” OR “atezolizumab” OR “MPDL 3280a” OR “avelumab” OR “AMP 224” OR “toripalimab” OR “camrelizumab” OR “SHR-1210” OR “sintilimab” OR “tislelizumab” OR “penpulimab” OR “zimberelimab” OR “envafolimab” OR “cemiplimab” AND “stomach neoplasms” OR “esophageal neoplasms” OR “gastric cancer” OR “esophageal cancer” OR “gastro-oesophageal junction cancer”. To avoid omission of any latently relevant research, the references to the primary articles and pertinent reviews were manually examined as well.

### Inclusion and exclusion criteria

The inclusion criteria were as follows: (Sung et al., 2021) RCTs were included in the study; (Ajani et al., 2016) patients suffering from either advanced esophageal, gastroesophageal junction or gastric cancer; (Van Cutsem et al., 2016) studies in which patients in the intervention group received PD-1/PD-L1 checkpoint inhibitors as a monotherapy or combined with other therapies (immunotherapy, chemotherapy, targeted therapy, and radiotherapy), while patients in the control group received placebo or other therapies that did not include PD-1/PD-L1 inhibitors; (Bray et al., 2018) study in which efficacy data of overall survival (OS), progression-free survival (PFS) was available.

The exclusion criteria were as follows: (Sung et al., 2021) articles not published in English; (Ajani et al., 2016) studies that fell within the category of adjuvant therapy or neoadjuvant therapy; (Van Cutsem et al., 2016) articles that were duplicated (the latest published or most complete article would be chosen for inclusion); (Bray et al., 2018) articles that were study protocols or did not report any relevant outcomes; (Zou et al., 2016) articles in which unable to access the full text or extract available data were not available.

### Data extraction

Data from each study were extracted by two investigators independently in the light of a pre-designed data extraction table. And any discrepancies were ironed out through third-party arbitration. The following information was extracted: Author, year of publication, country, trial phase, therapy lines, tumour type, sample size, therapeutic schedule, median follow up duration, participants’ characteristics (e.g., mean age, sex ratio, eastern cooperative oncology group (ECOG) performance status, geographic region), trial registration number, and data on survival outcomes. Furthermore, the data were also collected for the pre-defined subgroups listed below: therapy lines, therapeutic schedules, tumour types, age, ECOG performance status, sex, geographical region, PD-L1 expression status.

### Quality evaluation and outcome measures

The Cochrane Collaborative Risk of Bias Assessment Tool was employed to appraise the potential risk of bias in RCTs (Higgins et al., 2011), which contained seven domains: random sequence generation, allocation concealment, blinding of participants and personnel, blinding of outcome assessment, incomplete outcome data, selective reporting and other source of bias. And the risk was divided into three levels: high risk, unclear risk, and low risk. The results showed that all of the studies included in the analysis had acceptable quality.

The primary efficacy objective of this meta-analysis was to determine the impact of PD-1/PD-L1 inhibitors on OS and PFS in patients with advanced esophageal, gastroesophageal junction or gastric cancer, as evaluated by the interaction hazard ratio (HR).

### Statistical analysis

By using Stata 12.0 software, the distinct data extracted in each included study were combined, so as to evaluate the efficacy of PD-1/PD-L1 inhibitors. The effect size was calculated as HR with 95% confidence interval (CI). For the total sample, the heterogeneity test was examined using the Cochran chi-squared and quantified using the inconsistency test (I2). According to the Cochrane Manual and study characteristics (Higgins et al., 2003), I2 values of 0%–30% showed mild or insignificant heterogeneity, 30%–70% revealed moderate heterogeneity, and 70%–100% indicated high or substantial heterogeneity. Considering the uncertainty of heterogeneity and the complexity of control conditions between included studies, a random-effects (RE) model was applied to meta-analysis. All probabilities (*p* values) of data were two-sided, with *p* < 0.05 considered as statistically significant.

To detect the latent variables leading to sources of heterogeneity, pre-specified subgroup analyses were executed. When included articles ≥10, the publication bias was assessd using Begg’s weighted regression test with significance set at *p* < 0.1 (Shen et al., 2019). Moreover, through excluding each study successively, the sensitivity analysis were performed as well.

## Results

### Study selection

**FIGURE 1:**
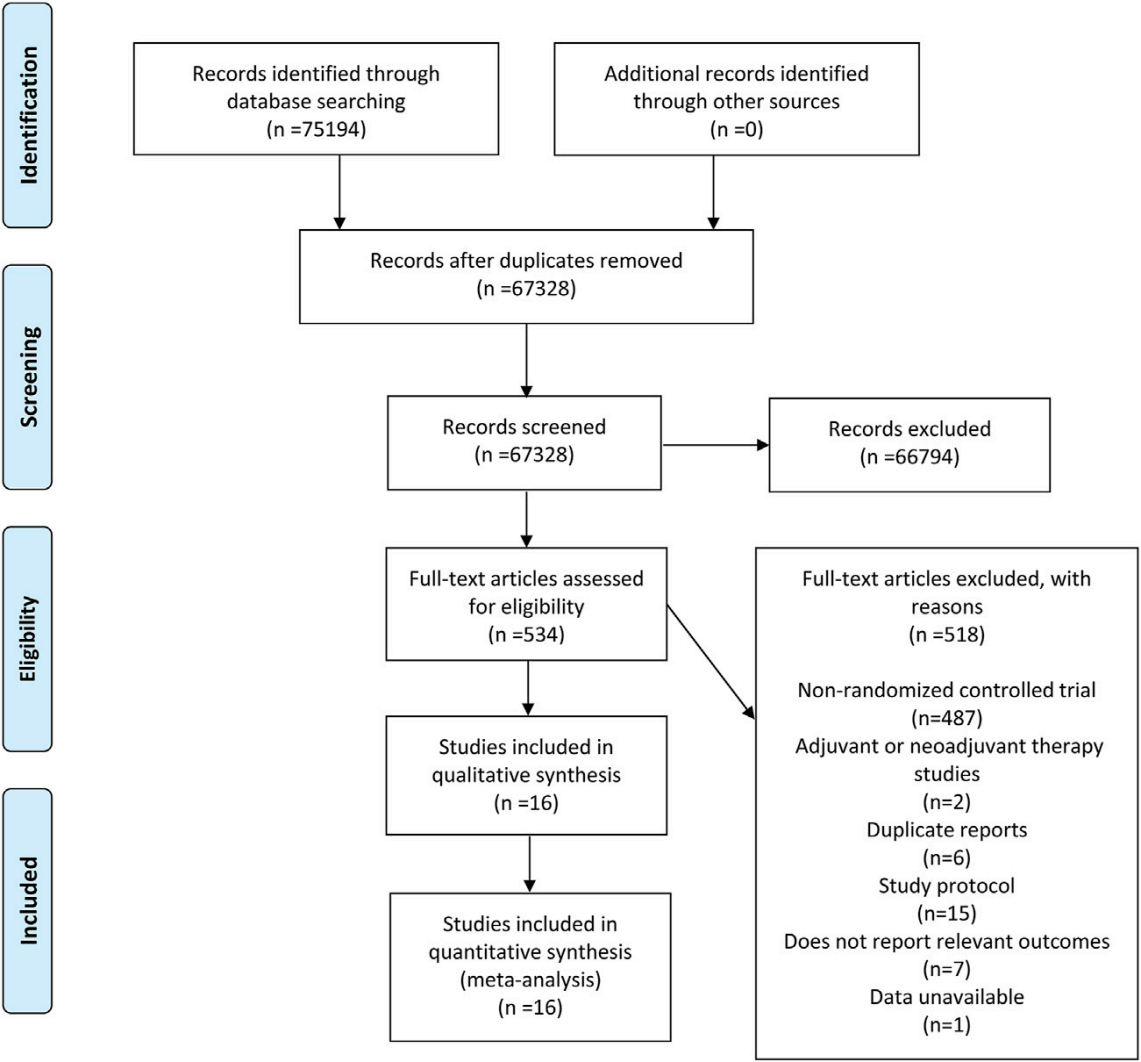
Flow diagram describing inclusion and exclusion criteria.

A total of 75,194 related records were screened following the database search. After removal of 7,866 duplicate studies, 67,328 studies were ruled out since their title and abstract did not match the inclusion criteria. Next, after reviewing and evaluating the full text of the remaining 534 studies, 487 articles were removed as the result of non-RCT, 15 study protocols were ruled out, and 2 were excluded due to adjuvant or neoadjuvant therapy studies. Furthermore, six studies were eliminated because of duplicate reports, seven articles were excluded owing to the fact that they failed to report relevant outcomes. In virtue of data unavailable, one trail was also removed. Ultimately, 16 studies (Bang et al., 2018; Huang et al., 2020; Kojima et al., 2020; Shitara et al., 2020; Boku et al., 2021; Janjigian et al., 2021; Luo et al., 2021; Moehler et al., 2021; Sun et al., 2021; Chung et al., 2022; Doki et al., 2022; Fuchs et al., 2022; Kang et al., 2022; Wang et al., 2022) were included in conformity to inclusion and exclusion criteria, the flow chart for the selection procedure and specific identification is manifested in Figure 1.

### Study characteristics and quality assessment

The fundamental characteristics of 16 studies demonstrating the efficacy of PD-1/PD-L1 inhibitors in patients suffered from advanced esophageal, gastroesophageal junction or gastric cancers are shown in Table 1; Supplementary Table S1. These studies comprised a total of 9,304 patients, including 4,865 in the experimental group and 4,439 in the control group. Among them, 15 studies were in phase III except one in phase II. The tumor types were comprised of esophageal, gastroesophageal junction and gastric cancer. For therapeutic schedules, eight studies (Shitara et al., 2020; Boku et al., 2021; Janjigian et al., 2021; Luo et al., 2021; Sun et al., 2021; Doki et al., 2022; Kang et al., 2022; Wang et al., 2022) included PD-1/PD-L1 inhibitors combined with chemotherapy *versus* chemotherapy, nine studies (Bang et al., 2018; Huang et al., 2020; Kojima et al., 2020; Shitara et al., 2020; Moehler et al., 2021; Chung et al., 2022; Fuchs et al., 2022; Okada et al., 2022; Xu et al., 2022) contained PD-1/PD-L1 inhibitor monotherapy *versus* chemotherapy, while one study (Boku et al., 2021) involved PD-1/PD-L1 inhibitor monotherapy *versus* placebo [one study was a three-arm clinical trial, including the group of PD-1/PD-L1 inhibitors combined with chemotherapy, the group of PD-1/PD-L1 inhibitors monotherapy and chemotherapy group (Shitara et al., 2020)]. The median follow-up time varied from 4.1 to 17.45 weeks. The detailed quality assessments, based on the Cochrane Collaboration’s tool, of the 16 studies are shown in Supplementary Figures S1, S2. Due to their open-label design, most of the trials had a high risk of performance bias. Other domains were assured with reasonably low risk based on the inclusion of high-quality RCTs. The overall quality was in accord with the meta-analysis criteria.

**TABLE 1:** The characteristics of studies included in this meta-analysis.

Author, year	Country	Phase	Line	Tumour	Sample size		Interventions		Median follow-up (weeks)	
					Experimental	Control	Experimental	Control		
Boku, 2021	USA	III	2L, 3L	GEJ, G	330	163	Nivolumab	Placebo	5.3	4.1
Janjigian, 2021	USA	III	1L	E, GEJ, G	789	792	Nivolumab + Chemotherapy	Chemotherapy	13.8	11.6
Kang, 2022	South Korea	III	1L	GEJ, G	362	362	Nivolumab + Chemotherapy	Placebo + Chemotherapy	17.45	17.15
Shitara, 2020	Spain	III	1L	GEJ, G	257; 256	250	Pembrolizumab + Chemotherapy; Pembrolizumab	Placebo + Chemotherapy	12.5^a^, 12.3^c^; 10.6^a^, 17.4^c^	11.1^a^, 10.8^c^
Sun, 2021	South Korea	III	1L	E, GEJ	373	376	Pembrolizumab + Chemotherapy	Placebo + Chemotherapy	12.4	9.8
Doki, 2022	Japan	III	1L	E	321	324	Nivolumab + Chemotherapy	Chemotherapy	13.2	10.7
Wang, 2022	China	III	1L	E	257	257	Toripalimab + Chemotherapy	Placebo + Chemotherapy	17	11
Luo, 2021	China	III	1L	E	298	298	Camrelizumab + Chemotherapy	Placebo + Chemotherapy	15.3	12.0
Chung, 2022	South Korea	III	2L	GEJ, G	47	47	Pembrolizumab	Chemotherapy	8	8
Fuchs, 2022	USA	III	2L	GEJ, G	294	296	Pembrolizumab	Chemotherapy	9.1^a^, 10.4^b^,^c^	8.3^a^,^b^, 10.8^c^
Bang, 2018	South Korea	III	3L	GEJ, G	185	186	Avelumab + BSC	Chemotherapy + BSC	4.6	5.0
Moehler, 2021	Germany	III	1L	GEJ, G	249	250	Avelumab	Chemotherapy + BSC	10.4	10.9
Kojima, 2020	Japan	III	1L	E, GEJ	314	314	Pembrolizumab	Chemotherapy	7.1	7.1
Huang, 2020	China	III	2L	E	228	220	Camrelizumab	Chemotherapy	8.3	6.2
Okada, 2022	Japan	III	2L	E	210	209	Nivolumab	Chemotherapy	10.9	8.5
Xu, 2022	China	II	2L	E	95	95	Sintilimab	Chemotherapy	7.2	6.2

1L, first line; 2L, second line; 3L, third line; E, esophageal cancer; GEJ, gastroesophageal junction cancer; G, gastric cancer; BSC, best supportive care; PD-L1, programmed death ligand-1; CPS, PD-L1 combined positive score.

a Median follow-up duration in patients with PD-L1 CPS ≥ 1.

b Median follow-up duration in patients with PD-L1 CPS ≥ 5.

c Median follow-up duration in patients with PD-L1 CPS ≥ 10.

### Overall survival outcomes

**FIGURE 2:**
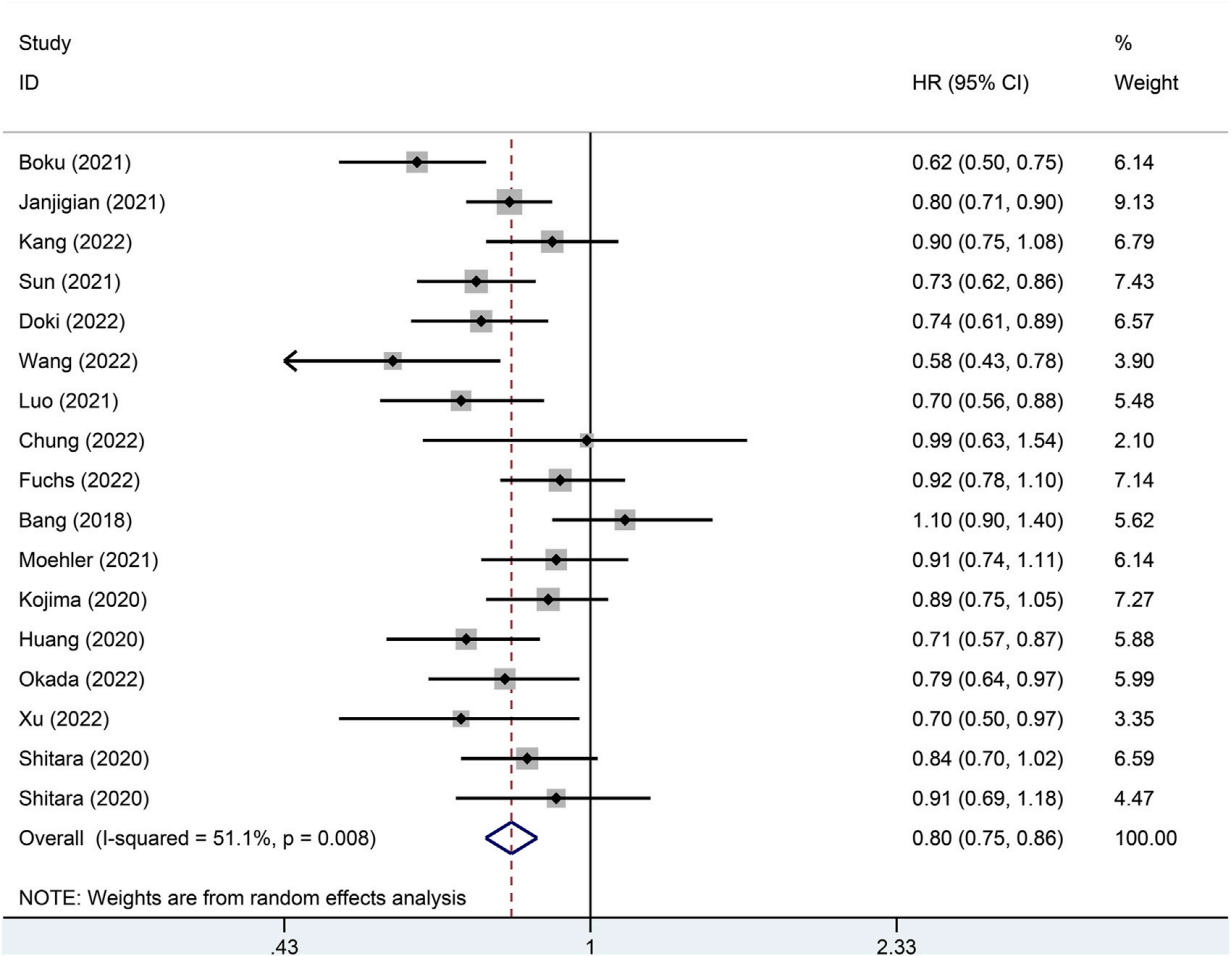
Forest plot of the *p* value for the overall survival in the general population.

Pooled analysis of the 16 studies for OS showed that patients treated with PD-1/PD-L1 inhibitors had longer OS compared with controls (HR = 0.80; 95% CI: 0.75–0.86; I2 = 51.1%; *p* < 0.001) (Figure 2), indicating that PD-1/PD-L1 inhibitors (monotherapy or combined with chemotherapy) could prolong the OS of esophageal, gastroesophageal junction and gastric cancer patients.

In subgroup analysis, when the line of therapy was divided into first-line therapy and non-first-line therapy, the results indicated that the OS of patients who received PD-1/PD-L1 inhibitors was significantly longer than controls in both first-line (HR = 0.80; *p* < 0.001) and non-first-line (HR = 0.81; *p* = 0.010) treatments. In regards to subgroup analyses of therapeutic schedules, patients in the PD-1/PD-L1 inhibitors group had a longer OS than controls, irrespective of whether they were treated with PD-1/PD-L1 inhibitors combined with chemotherapy (HR = 0.75; *p* < 0.001) or PD-1/PD-L1 inhibitor monotherapy (HR = 0.87; *p* = 0.003). However, the results also showed that the curative effect of PD-1/PD-L1 inhibitors combined with chemotherapy seemed to be better than that of PD-1/PD-L1 inhibitor monotherapy.

To investigate the effects of different tumor types on the efficacy of PD-1/PD-L1 inhibitors, relevant studies were categorized into esophageal cancer (HR = 0.75; *p* < 0.001), gastroesophageal junction cancer (HR = 0.87; *p* = 0.040) and gastric cancer (HR = 0.87; *p* = 0.019) (Table 2). Pooled analysis showed that compared with the control group, the OS of patients treated with PD-1/PD-L1 inhibitors was improved in all three tumor types, with esophageal cancer benefiting more from PD-1/PD-L1 inhibitors (HR = 0.75; *p* < 0.001). Among them, when the esophageal cancer cases were grouped into adenocarcinoma and squamous cell carcinoma subgroups based on histological classification, compared with the control group, patients with esophageal squamous cell carcinoma were found to have prolonged OS (HR = 0.75; *p* < 0.001), while no statistical difference was discovered in patients with esophageal adenocarcinoma between the experimental group and the control group (*p* = 0.679). In regard to gastric cancer, when the patients were classified into intestinal and diffuse types based on the Lauren classification, the results showed that PD-1/PD-L1 inhibitors could improve the OS of both intestinal (HR = 0.80; 95% CI: 0.71–0.92) and diffuse (HR = 0.84; 95% CI: 0.72–0.99) types compared with the control group. The pooled effects are as emerged in Table 2.

**TABLE 2:** Subgroup analysis of overall survival.

Subgroup analysis		No. of studies	HR	95%CI	p	Heterogeneity (I2) (%)
Line of therapy	First line	9	0.80	0.75–0.86	<0.001	33.2
	Non-first line	7	0.81	0.69–0.95	0.010	68.8
Therapeutic schedules	PD-1/PD-L1 + Chemotherapy vs. Chemotherapy	8	0.75	0.68–0.82	<0.001	46.0
	PD-1/PD-L1 vs. Chemotherapy	9	0.87	0.80–0.96	0.003	30.0
Tumour types	E	9	0.75	0.70–0.81	<0.001	3.4
	GEJ	7	0.87	0.76–0.99	0.040	0
	G	7	0.87	0.77–0.98	0.019	57.2
Histological type (E/GEJ)	Adenocarcinoma	2	0.92	0.61–1.38	0.679	73.3
	Squamous cell carcinoma	2	0.75	0.65–0.86	<0.001	0
Lauren classification (G/GEJ)	Intestinal type	5	0.80	0.71–0.92	0.001	12.9
	Diffuse type	5	0.84	0.72–0.99	0.032	43.7
Age	< 65 years	15	0.81	0.75–0.87	<0.001	20.4
	≥ 65 years	15	0.80	0.73–0.88	<0.001	32.7
ECOG performance status	0	14	0.82	0.73–0.91	<0.001	39.2
	1	14	0.78	0.71–0.86	<0.001	46.9
Sex	Male	15	0.78	0.72–0.84	<0.001	43.4
	Female	15	0.92	0.84–1.02	0.120	0
Geographical region	Asia	15	0.75	0.68–0.82	<0.001	44.2
	Non-Asia	10	0.90	0.83–0.97	0.006	17.2
TPS	TPS < 1%	8	0.89	0.80–0.99	0.030	3.0
	TPS ≥ 1%	9	0.69	0.58–0.83	<0.001	35.0
	TPS < 5%	4	0.78	0.68–0.89	<0.001	0
	TPS ≥ 5%	4	0.62	0.52–0.74	<0.001	0
	TPS < 10%	5	0.77	0.68–0.86	<0.001	0
	TPS ≥ 10%	4	0.66	0.52–0.83	<0.001	0
CPS	CPS < 1	4	0.91	0.72–1.15	0.416	0
	CPS ≥ 1	7	0.77	0.72–0.84	<0.001	0
	CPS < 5	2	0.87	0.70–1.08	0.221	29.7
	CPS ≥ 5	3	0.71	0.63–0.79	<0.001	0
	CPS < 10	5	0.86	0.74–0.99	0.038	22.3
	CPS ≥ 10	8	0.68	0.61–0.77	<0.001	0

HR, hazard ratio; CI, confidence interval; ECOG, Eastern Cooperative Oncology Group; PD-1, programmed death-1 receptor; PD-L1, programmed death ligand-1; TPS, PD-L1 tumour proportion score; CPS, PD-L1 combined positive score; E, esophageal cancer; GEJ, gastroesophageal junction cancer; G, gastric cancer.

For age subgroup analysis, 65 years was used as the threshold. The analysis results demonstrated that compared with the control group, patients receiving PD-1/PD-L1 inhibitors benefited from longer OS in both <65 years (HR = 0.81; 95% CI: 0.75–0.87) and ≥65 years (HR = 0.80; 95% CI: 0.73–0.88) groups. For subgroup analysis on ECOG performance status 0 or 1, the results showed that compared with the control group, the OS of patients treated with PD-1/PD-L1 inhibitors was also prolonged in the ECOG performance status 0 (HR = 0.82; 95% CI: 0.73–0.91) and status 1 (HR = 0.78; 95% CI: 0.71–0.86) groups. Furthermore, it was worth noting that when subgroup analysis of gender was performed, compared with the control group, treatment with PD-1/PD-L1 inhibitors in the male subgroup was associated with significant clinical effectiveness in OS improvement (HR = 0.78; *p* < 0.001), while no statistical difference was observed in the female segment between the two gruops (*p* = 0.120). In terms of geographical region, Asian (HR = 0.75; 95% CI: 0.68–0.82) and non-Asian (HR = 0.90; 95% CI: 0.83–0.97) patients demonstrated longer OS with PD-1/PD-L1 inhibitors compared with controls. However, the results also showed that the Asian population tended to have superior treatment efficacy with PD-1/PD-L1 inhibitors therapy compared with non-Asian patients. Table 2 displays the relevant outcome values.

To determine the impact of PD-L1 expression on treatment efficacy, PD-L1 tumor proportion score (TPS) and PD-L1 combined positive score (CPS) was chosen as the evaluation indicators. When 1% of TPS was used as the cutoff, the results showed that the OS of patients (esophageal cancer, gastroesophageal junction cancer and gastric cancer) in the TPS ≥1% group (HR = 0.69; 95% CI: 0.58–0.83) and TPS <1% group (HR = 0.89; 95% CI: 0.80–0.99) was superior to the control group, with patients of TPS ≥1% benefiting more from PD-1/PD-L1 inhibitors. When a TPS of 5% was used as the threshold, the results showed that patients with TPS <5% (HR = 0.78; 95% CI: 0.68–0.89) and TPS ≥5% (HR = 0.62; 95% CI: 0.52–0.74) had better OS compared with controls and that the TPS ≥5% group demonstrated greater OS benefit. Further, when a TPS of 10% was used as the cutoff point, compared with the control group, significant improvements in OS were observed in both groups with TPS <10% (HR = 0.77; 95% CI: 0.68–0.89) and TPS ≥10% (HR = 0.66; 95% CI: 0.52–0.83), with TPS ≥10% group showing greater benefit with PD-1/PD-L1 inhibitors compared with the TPS <10% group.

When CPS <1 or ≥1 was used as the threshold, the results showed that patients with CPS ≥1 (HR = 0.77 *p* < 0.001) had greater OS prolongation with PD-1/PD-L1 inhibitors compared with controls, while no statistical difference was found between patients with CPS <1 among the experimental and control group (*p* = 0.416). For CPS subgroups <5 or ≥5, compared to the control group, a significant prolongation in OS was observed in patients with CPS ≥5 (HR = 0.71; *p* < 0.001), while no statistical difference was detected in the patients with CPS <5 between the experimental and control groups (*p* = 0.221). Lastly, for CPS subgroups <10 or ≥10, a greater improvement in OS with PD-1/PD-L1 inhibitors was observed for patients with CPS ≥10 (HR = 0.68; 95%CI: 0.61–0.77) and CPS <10 (HR = 0.86; 95%CI: 0.74–0.99) compared with the control group, with the CPS ≥10 group demonstrating more benefit from PD-1/PD-L1 inhibitors. The results of the pooled effects are shown in Table 2.

### Progression-free survival outcomes

Altogether, there were 15 RCTs investigating PFS. A high heterogeneity between the included studies was observed, and the pooled PFS outcomes indicated no significant difference between the PD-1/PD-L1 inhibitor and control group (HR = 0.89; 95% CI: 0.75–1.06; I2 = 91.1%; *p* = 0.185). The pooled effects of the overall cohort of the 15 studies are shown in Table 3.

**TABLE 3:** Overall effects and subgroup analysis of progression-free survival.

Subgroup analysis		No. of studies	HR	95%CI	p	Heterogeneity (I2) (%)
Overall		15	0.89	0.75–1.06	0.185	91.1
Line of therapy	First line	9	0.83	0.68–1.01	0.062	91.3
	Non-first line	6	1.02	0.72–1.46	0.901	91.7
Therapeutic schedules	PD-1/PD-L1 + Chemotherapy vs. Chemotherapy	8	0.68	0.62–0.76	<0.001	60.0
	PD-1/PD-L1 vs. Chemotherapy	8	1.18	0.94–1.47	0.146	86.8
Tumour types	E	8	0.78	0.64–0.95	0.015	86.4
	GEJ	2	0.89	0.44–1.81	0.755	65.8
	G	2	1.16	0.44–3.09	0.759	95.9
Age	< 65 years	5	0.75	0.50–1.14	0.179	91.9
	≥ 65 years	5	0.74	0.55–1.00	0.049	74.9
ECOG performance status	0	5	0.73	0.45–1.19	0.208	89.0
	1	5	0.76	0.55–1.04	0.088	87.5
Sex	Male	5	0.71	0.51–0.98	0.037	90.1
	Female	5	0.98	0.57–1.68	0.932	83.8
Geographical region	Asia	8	0.84	0.61–1.16	0.301	92.5
	Non-Asia	2	0.89	0.52–1.53	0.681	78.6
TPS	TPS < 1%	3	0.93	0.47–1.86	0.836	94.6
	TPS ≥ 1%	4	0.90	0.50–1.60	0.720	83.2
CPS	CPS <	2	0.86	0.65–1.14	0.297	4.4
	CPS ≥ 1	5	0.94	0.69–1.27	0.671	92.6
	CPS < 10	2	0.68	0.48–0.97	0.031	68.2
	CPS ≥ 10	5	0.72	0.58–0.91	0.005	67.4

HR, hazard ratio; CI, confidence interval; ECOG, Eastern Cooperative Oncology Group; PD-1, programmed death-1 receptor; PD-L1, programmed death ligand-1; TPS, PD-L1 tumour proportion score; CPS, PD-L1 combined positive score; E, esophageal cancer; GEJ, gastroesophageal junction cancer; G, gastric cancer.

Regarding subgroup analysis for therapeutic schedules, in comparison with the control group, PD-1/PD-L1 inhibitor combined with chemotherapy was found to markedly prolong PFS (HR = 0.68; *p* < 0.001), whereas the difference was not statistically significant with PD-1/PD-L1 inhibitor monotherapy between the experimental and control group (*p* = 0.146). Among all the subgroups of tumor types, consisting of esophageal cancer (HR = 0.78; *p* = 0.015), gastroesophageal junction cancer (*p* = 0.755) and gastric cancer (*p* = 0.759), when compared with the control group, only esophageal cancer patients had substantial improvement in PFS. Subgroup analysis based on therapy lines, ECOG performance status and geographical region showed that compared with controls, treatment with PD-1/PD-L1 inhibitors did not improve the PFS of patients with first-line therapy (*p* = 0.062) or non-first-line therapy (*p* = 0.901), ECOG performance status of 0 (*p* = 0.208) or 1 (*p* = 0.088) and Asian population (*p* = 0.301) or non-Asian population (*p* = 0.681). For patients' age, the analysis revealed that those aged ≥65 years old (HR = 0.74; *p* = 0.049) had longer PFS than controls, while no significant difference was observed in patients aged <65 years old (*p* = 0.179) among the two groups. In addition, consistent with the above analysis of OS in terms of gender, when the subgroups were distinguished by gender, compared with the control group, therapy with PD-1/PD-L1 inhibitors was associated with significant PFS improvement in the experimental group of male patients (HR = 0.71; *p* = 0.037), while no significant statistical difference was observed in female patients among the experimental and control group (*p* = 0.932). For the influence of PD-L1 expression on efficacy, when TPS was chosen as the predictive marker, the differences were not statistically significant for TPS between the experimental and control group, nor for groups of TPS <1% (*p* = 0.863) or TPS ≥1% (*p* = 0.720). When CPS was used as the predictive marker, compared with the control group, a significant improvement with PD-1/PD-L1 inhibitors on PFS was observed only in patients with CPS <10 (HR = 0.68; *p* = 0.031) or CPS ≥10 (HR = 0.72; *p* = 0.005), while no statistical difference was found in both groups of CPS <1 (*p* = 0.297) or CPS ≥1 (*p* = 0.671) between the experimental group and the control group. The pooled effects in all subgroups are shown in Table 3.

### Effect of overall survival in subgroups of age and sex

To further explore the influence of age and gender on the clinical efficacy of PD-1/PD-L1 inhibitors, variables including lines of therapy, therapeutic schedules and tumor types were merged for pooled analysis. The results showed that both subgroups of <65 or ≥65 years demonstrated significantly prolonged OS with PD-1/PD-L1 inhibitors compared with the control group. The detailed data are shown in Table 4.

**TABLE 4:** Subgroup analysis of overall survival in age.

	Subgroup analysis	HR	95%CI	*p*
< 65 years	First line	0.80	0.74–0.86	<0.001
	Non-first line	0.82	0.68–0.98	0.028
≥ 65 years	First line	0.81	0.73–0.90	<0.001
	Non-first line	0.77	0.62–0.96	0.019
< 65 years	PD-1/PD-L1 + Chemotherapy vs. Chemotherapy	0.77	0.71–0.84	<0.001
	PD-1/PD-L1 vs. Chemotherapy	0.84	0.75–0.95	0.004
≥ 65 years	PD-1/PD-L1 + Chemotherapy vs. Chemotherapy	0.77	0.67–0.88	<0.001
	PD-1/PD-L1 vs. Chemotherapy	0.83	0.72–0.95	0.009
< 65 years	E + GEJ	0.75	0.68–0.82	<0.001
	G + GEJ	0.87	0.77–0.98	0.022
≥ 65 years	E + GEJ	0.74	0.66–0.84	<0.001
	G + GEJ	0.87	0.74–1.03	0.100

However, when the sex subgroup was analyzed, compared with the control group, the data revealed a substantial improvement in OS for males in lines of therapy, therapeutic schedules and tumor types, while females had no statistical difference in any of the investigated categories between the experimental and control group. Table 5 comprises the detailed data.

**TABLE 5:** Subgroup analysis of overall survival in sex.

	Subgroup analysis	HR	95%CI	*p*
Male	First line	0.77	0.71–0.84	<0.001
	Non-first line	0.78	0.67–0.91	0.002
Female	First line	0.92	0.82–1.03	0.153
	Non-first line	0.89	0.66–1.21	0.461
Male	PD-1/PD-L1 + Chemotherapy vs. Chemotherapy	0.73	0.66–0.81	<0.001
	PD-1/PD-L1 vs. Chemotherapy	0.82	0.74–0.90	<0.001
Female	PD-1/PD-L1 + Chemotherapy vs. Chemotherapy	0.90	0.79–1.03	0.141
	PD-1/PD-L1 vs. Chemotherapy	0.94	0.79–1.11	0.469
Male	E + GEJ	0.73	0.66–0.80	<0.001
	G + GEJ	0.84	0.75–0.95	0.004
Female	E + GEJ	0.87	0.70–1.06	0.171
	G + GEJ	0.99	0.87–1.14	0.906

### Publication bias and sensitivity analysis

To evaluate publication bias of OS in the general gastroesophageal cancer patients who was treated with PD-1/PD-L1 inhibitors, the quantificational Begg’s test was employed. And the *p* value of Begg’s funnel plots was 0.509, indicating no potential publication bias among the included articles on HR for OS. The related data was shown in Supplementary Figures S3. Apart from that, sensitivity analysis was also performed to appraise the stability of HR for OS by omitting each study in sequence successively, and the results confirmed the excellent stability. The relevant data was comprised in Supplementary Figures S4.

## Discussion

As a negative costimulatory receptor, PD-1 is expressed on activated T-cells and binds to the PD-L1 ligand to downregulate T-cell-mediated immune responses (Nishimura et al., 1999; Freeman et al., 2000; Greenwald et al., 2005). Therefore, due to the activation of the PD-1 signaling pathway and the overexpression of PD-L1 in tumor cells, malignant tumors can escape immune surveillance (Topalian et al., 2012; Comprehensive molecular characterization of gastric, 2014). Based on the above theoretical basis, blocking the related signal pathways with PD-1/PD-L1 inhibitors may restore the immune activity of T cells (Topalian et al., 2012; Comprehensive molecular characterization of gastric, 2014). In terms of pathophysiology, gastroesophageal cancer is not considered an immune-related type of cancer in the conventional sense. Nonetheless, several studies have revealed that the number of gastroesophageal tumor-infiltrating lymphocytes could be associated with the progression of the tumor and patients' prognosis (Short et al., 2017; Zhu et al., 2017). Based on which PD-1/PD-L1 inhibitors have been investigated as a therapeutic strategy for advanced gastroesophageal cancer patients.

This present systematic review and meta-analysis was performed to investigate the clinical efficacy of PD-1/PD-L1 inhibitors on advanced gastroesophageal cancer as well as the relationship between clinicopathological features and the curative effects of PD-1/PD-L1 inhibitors. Consistent with the results of previous meta-analyses (Chen et al., 2021; Formica et al., 2021; Oh et al., 2021), we found that PD-1/PD-L1 inhibitors (monotherapy or combined with chemotherapy) were associated with a prolongation of OS in patients with esophageal cancer, gastroesophageal junction cancer and gastric cancer, and it should be emphasized that no significant improvement in PFS was observed. Although both OS and PFS are regarded as important survival outcomes of response efficacy in anticancer treatment, the link between the two has yet to be fully proved. OS is defined as the time from randomization to death from any cause, with the outcome measure being time to death. Due to longer follow-up duration, patients' OS is more susceptible to further treatment, cross-treatment, and other diseases. Nonetheless, for clinical oncology trials, since the goal of cancer treatment is to prolong survival, OS remains the gold standard for reflecting the endpoint of curative effect in cancer treatment (Fiteni et al., 2014; Sarac et al., 2019). Compared with OS, the follow-up duration of PFS is significantly shortened and has been used as a favorable surrogate endpoint for OS in different cancer therapies such as chemotherapy, radiotherapy, targeted therapy, etc. However, in the field of immunotherapy, whether PFS performs better than traditional response evaluation criteria such as OS in evaluating efficacy or clinical benefits has not been verified in clinical trial settings (Zhu et al., 2021). Besides, several studies have demonstrated that advanced esophageal cancer patients with biomarker differences including no 11q13 chromosomal amplification, high tumor mutation burden (TMB), and microsatellite instability-high (MSI-H) tended to have greater OS rather than PFS benefit from PD-1/PD-L1 inhibitors (Mocellin et al., 2001; Greally and Ku, 2018; Lu et al., 2021). As a result, OS might be a better curative effect endpoint in evaluating the clinical efficacy of PD-1/PD-L1 inhibitors in advanced gastroesophageal cancer to some extent.

In line with recent studies, ICIs therapy might still be beneficial in patients with various malignancies, including gastroesophageal cancer. However, the low response rates in patients with advanced cancer and the reduced incidence of immune-related adverse events in unresponsive patients remain the major obstacles to treating cancer with immune checkpoint therapy (Darvin et al., 2018). Therefore, to facilitate patient selection and decision-making of PD-1/PD-L1 inhibitors therapy, further research into highly reliable predictive markers is required. The subgroup analysis of this present study showed that patients with esophageal cancer, gastroesophageal junction cancer and gastric cancer could benefit from PD-1/PD-L1 inhibitors treatment in both first-line and non-first-line therapy. Previously, although there have been numerous studies on the usage of PD-1/PD-L1 inhibitors in the treatment of gastroesophageal cancer worldwide, none has been officially approved by the FDA. However, it is worth noting that there have been some recent breakthroughs in this field. In May 2021, the FDA approved nivolumab in combination with chemotherapy as first-line therapy for advanced or metastatic esophageal adenocarcinoma, gastroesophageal junction cancer and gastric cancer, representing the first time a new treatment for stage II/III gastroesophageal cancer has been approved apart from chemotherapeutics (Weadick et al., 2022). The results of this present study also provide some evidence for more PD-1/PD-L1 inhibitors to be assessed as first-line treatment in gastroesophageal cancer. Further, it was reported that because patients receiving first-line treatment might have better performance status than those receiving non-first-line treatment, they might respond more favorably to immunotherapy (Reck et al., 2019). Nevertheless, our study showed consistent efficacy between the first-line and non-first-line treatments. As for ECOG performance status, our results demonstrated that it might not be a key factor affecting the efficacy of PD-1/PD-L1 inhibitors, as improvements in OS were observed in both subgroups of ECOG performance status.

For therapeutic schedules, some studies demonstrated that PD-1/PD-L1 inhibitors in combination with chemotherapy could promote tumor antigen cross-presentation and up-regulate the expression of major histocompatibility complex (MHC) class I antigens (Serrano et al., 2001). In addition, in the presence of interleukin (IL)-2, IL-5 and other cytokines, increasing the activation of CD8^+^ T-cells with PD-1/PD-L1 inhibitors combined with chemotherapy was shown to further improve its tumor-killing ability compared with chemotherapy (Serrano et al., 2001). Hence, consistent with previous clinical studies (Janjigian et al., 2021; Doki et al., 2022; Kang et al., 2022), our results also support the combination of PD-1/PD-L1 inhibitors with chemotherapy to potentially prolong the patients' OS and PFS.

Gastroesophageal cancer is an umbrella term for tumors with extensive heterogeneity among different histologic types and tumor topographical locations. Anatomically, it can be subdivided into esophageal cancer, gastroesophageal junction cancer and gastric cancer. Our results showed that PD-1/PD-L1 inhibitors significantly improved the OS and PFS of esophageal cancer patients compared with those having gastroesophageal junction and gastric cancer. For gastroesophageal junction cancer, previous studies showed that in terms of clinical and pathological features, gastroesophageal junction cancer in Chinese patients was mainly associated with proximal gastric cancer, compared with distal esophageal cancer in American patients (Huang et al., 2011; Chevallay et al., 2018). Therefore, the therapeutic schedules for advanced gastroesophageal junction cancer remain controversial. According to our analysis, PD-1/PD-L1 inhibitors had similar OS improvement in gastroesophageal junction cancer, gastric cancer, and esophageal cancer. In regard to the histological subtypes of esophageal cancer, the Japanese Endoscopy Society and The Cancer Genomic Atlas (TCGA) categorizes it as adenocarcinoma and squamous cell carcinoma (Japanese classification of gastric carcinoma, 2011; Comprehensive molecular characterization of gastric, 2014). For gastric cancer, adenocarcinoma accounts for 95% of all gastric cancer cases and is classified as intestinal (with intracellular junctions) or diffuse (without intracellular junctions) based on the Lauren classification (Lauren, 1965). Further, it was reported that the overexpression of PD-L1 might be related to better efficacy of PD-1/PD-L1 inhibitors (Vrána et al., 2018) and that the overexpression of PD-L1 was greater in esophageal squamous cell carcinoma than in esophageal adenocarcinoma and gastric adenocarcinoma (including intestinal and diffuse type) (Maoxi et al., 2021). According to our data analysis, this might be one of the reasons why esophageal squamous cell carcinoma demonstrated more effective treatment efficacy with PD-1/PD-L1 inhibitors compared with esophageal adenocarcinoma and gastric cancer.

Intriguingly, subgroup analysis on age demonstrated that PD-1/PD-L1 inhibitors could considerably prolong the OS of gastroesophageal cancer patients regardless of the patient’s age. In general, aging significantly impacts normal cells in the tumor microenvironment, causing frailty in older patients to limit their capacity to receive further treatment (Landre et al., 2020). However, after the variables including line of therapy, therapeutic schedules, and tumor types were merged with age for pooled analysis, significant potential curative effects with PD-1/PD-L1 inhibitors were observed in the <65 or ≥65 years subgroups. A previous meta-analysis targeted at age also revealed that in the first line treatment, PD-1/PD-L1 inhibitors were effective not only for patients aged <75 years, but also for patients aged >75 years (Landre et al., 2020). Thus, the upper age limit of PD-1/PD-L1 inhibitors might need to be re-assessed in the future. Due to the limited treatment options for advanced gastroesophageal cancer in the elderly, PD-1/PD-L1 inhibitors could be considered as a potentially effective treatment option.

The most notable finding of this study was that when subgroup analysis was performed on gender, the results illustrated that for gastroesophageal cancer patients, compared to chemotherapy, PD-1/PD-L1 inhibitors were associated with OS and PFS benefits in males but not in females (i.e., immunotherapy was more effective in males). When lines of therapy, therapeutic schedules and tumor types were pooled with sex for pooled analysis, the findings still supported this trend. Conforti et al. (2018) reported that PD-1/PD-L1 inhibitors could improve the OS of males and females with advanced or metastatic cancer, though male patients had a twofold reduction in mortality risk compared with females. The phenomenon might be interpreted by the fact that compared with males, the MHC-based presentation of driver mutations might be poorer in females; therefore, even if T-cells restore the anti-tumor immune activity under the action of PD-1/PD-L1 inhibitors, triggering the appropriate immune killing response might be more challenging in females than in males (Dinesh et al., 2010; Castro et al., 2020). Another reason might be closely related to the TMB, defined as the total number of nonsynonymous mutations detected per million bases. TMB has emerged as a biomarker for predicting the efficacy of PD-1/PD-L1 inhibitors in several cancers (Snyder et al., 2014; Rizvi et al., 2015). It was even reported that the TMB of men was higher than women in melanoma and non-small cell lung cancer, indicating that the tumor cells of male patients could be more antigenic and respond better to PD-1/PD-L1 inhibitors, resulting in superior curative effects (Gupta et al., 2015; Wang et al., 2019a; Wang et al., 2019b). Additionally, behavioral differences between males and females, such as a higher frequency of smoking and alcohol abuse in males, might also impact the human body’s internal environment, leading to sex differences in immune responses to a certain extent. However, recent studies have shown that a large number of androgens secreted by men could induce the exhaustion of CD8^+^ T-cell, thereby losing their anticancer activity (Kwon et al., 2022; Yang et al., 2022). Thus, blocking the androgen-androgen receptor axis could reshape the tumor microenvironment, facilitate the differentiation of effector T-cells and enhance the therapeutic effect of anti-PD-1 immune checkpoint blockade. Moreover, due to the gender imbalance in the study, the relationship between gender and PD-1/PD-L1 inhibitors treatment still needs to be further investigated.

With regard to geographical region, this study indicated that the efficacy of PD-1/PD-L1 inhibitors in the Asian gastroesophageal cancer population appeared to be better than that of the non-Asian population, which was consistent with the results of a previous meta-analysis (Formica et al., 2021). For non-Asian patients, risk factors such as obesity, smoking, and alcohol consumption are mostly responsible for the occurrence of gastroesophageal cancer (Chow et al., 1995; Lagergren et al., 1999; Wu et al., 2003; Hoyo et al., 2012; Lubin et al., 2012). However, in the Asian population, *Helicobacter pylori* (*H. pylori*) infection is regarded as a high-risk factor for gastric cancer and esophageal squamous cell carcinoma (Ye et al., 2004; Crew and Neugut, 2006; Rahman et al., 2014). Recent studies indicated that chronic *H. pylori* infection could induce gastric epithelial cells in patients with negative PD-L1 expression to express PD-L1 while causing local inflammation, reshaping the tumor microenvironment, and making local immune cells more likely to be activated to better respond to ICIs (Wu et al., 2010; Nishizuka et al., 2018; Koizumi et al., 2022). As a result, due to the high prevalence of *H. pylori* infection in Asian populations, compared with non-Asian populations, Asian populations may have greater efficacy for PD-1/PD-L1 inhibitors to a certain extent (Eusebi et al., 2014; Flores-Treviño et al., 2018).

This study also showed that although the OS of both TPS and CPS was prolonged with PD-1/PD-L1 inhibitors, TPS did not appear to adequately illustrate the relation between PD-L1 expression and treatment efficacy. When CPS <1, <5, and <10 were used, the results were statistically insignificant. Comparatively, TPS measures PD-L1 expression based on tumor ratio only (Dolled-Filhart et al., 2016), while CPS is defined as the ratio of all cells associated with PD-L1 expression to the total number of tumor cells. CPS has also been shown to be superior to the original TPS as a robust and reproducible PD-L1 scoring method after clinical verification (Kulangara et al., 2019; Weadick et al., 2022). Overall, CPS might outperform TPS in PD-L1 expression and treatment efficacy with PD-1/PD-L1 inhibitors in advanced gastroesophageal cancer.

In conclusion, this meta-analysis provides an update on the potential influencing factors such as treatment strategy, gender and PD-L1 expression in patients with gastroesophageal cancer treated with PD-1/PD-L1 inhibitors. Although earlier studies were performed on predictive markers, including gender and PD-L1 expression (Formica et al., 2021), however, due to the limited number of studies previously investigated, significant heterogeneity in the obtained results and discrepancies in different evaluation indicators of PD-L1 expression (i.e., TPS, CPS), an update on the significance of PD-1/PD-L1 inhibitors in gastroesophageal cancer was urgently needed. In this present study, more recent RCTs were included in the analyses, thereby providing new evidences and support for the clinical efficacy of PD-1/PD-L1 inhibitors in gastroesophageal cancer. Nevertheless, there were several limitations in this study that should be mentioned. First, the population covered in the study had an unbalanced gender ratio. Therefore, more studies should be conducted on the correlation between gender and PD-1/PD-L1 inhibitors efficacy in patients with gastroesophageal cancer. Second, although TMB and MSI-H were both biomarkers indicating better efficacy of PD-1/PD-L1 inhibitors, we cannot perform subgroup analysis of TMB and MS-H on account of unavailable data. Third, due to the limited number of studies, we were unable to perform subgroup analysis of other therapeutic schedules such as PD-1/PD-L1 inhibitors *versus* targeted therapy, PD-1/PD-L1 inhibitors *versus* radiotherapy, etc. Fourth, since few included studies further classified gastroesophageal junction cancer (e.g., Siewert classification or Nishi classification), we could not conclude the impact of PD-1/PD-L1 inhibitors on the efficacy of different types of gastroesophageal junction cancer.

## Conclusion

In conclusion, this meta-analysis demonstrates that PD-1/PD-L1 inhibitors (monotherapy or combined with chemotherapy) were associated with a prolongation of OS in patients with advanced gastroesophageal cancer, while there was no significant difference in the improvement of PFS. For clinicopathological features, evaluation indicators including therapeutic schedules, tumour types, histological type, sex, geographical region and PD-L1 expression status (TPS and CPS) seemed to be relevant to survival outcomes. Besides, therapy line, Lauren classification, age and ECOG performance status may not be the pivotal factors influencing the efficacy of PD-1/PD-L1 inhibitors. Based on considerations of relevant limitations, large sample RCTs are still needed to further confirm the study conclusion.

## Data Availability

The original contributions presented in the study are included in the article/Supplementary Material, further inquiries can be directed to the corresponding authors.

## Supplementary material

SUPPLEMENTARY FIGURE S1 Quality assessment of studies included in this meta-analysis (risk of bias graph).

SUPPLEMENTARY FIGURE S2 Quality assessment of studies included in this meta-analysis (Risk of bias summary).

SUPPLEMENTARY FIGURE S3 The *p* value of Begg’s funnel plots for publication bias.

SUPPLEMENTARY FIGURE S4 Sensitivity analysis of the hazard ratios of overall survival.

## Abbreviations

1L; first line; 2L, second line; 3L, third line; BSC, best supportive care; CI, confidence interval; CPS, PD-L1 combined positive score; E, esophageal cancer; ECOG, eastern cooperative oncology group; FDA, the Food and Drug Administration; G, gastric cancer; GEJ, gastroesophageal junction cancer; H. pylori, Helicobacter pylor; HR, hazard ratio; ICIs, immune checkpoint inhibitors; IL, interleukin; MHC, major histocompatibility complex; MSI-H, microsatellite instability-high; NA, no available; OS, overall survival; PD-1, programmed death-1 receptor; PD-L1, programmed death ligand-1; PFS, progression-free survival; RCTs, randomized controlled trials; RE, random-effects; TCGA, the Japanese Endoscopy society and The Cancer Genomic Atlas; TMB, tumor mutation burden; TPS, PD-L1 tumour proportion score.
