# Supplementary material for: Efficacy of PD-1/PD-L1 inhibitors in patients with advanced gastroesophageal cancer: An updated meta-analysis based on randomized controlled trials
Source: Front Pharmacol. 2022 Oct 25;13:1009254. doi: 10.3389/fphar.2022.1009254 (PMC9640921; doi:10.3389/fphar.2022.1009254)
Supplement: Supplementary file 1 [file Table1.docx]

**Supplementary Table 1.** The characteristics of studies included in this meta-analysis.

| Author, year | Trial registration number | Mean age (year) | | Experiment | | Control | | Experiment  (ECOG) | | Control (ECOG) | | Geographic region |
| --- | --- | --- | --- | --- | --- | --- | --- | --- | --- | --- | --- | --- |
|  |  | Experiment | Control | male | female | male | female | 0 | 1 | 0 | 1 |  |
| Boku, 2021 | NCT02267343 | 62 (54-69) | 61 (53-68) | 229 | 101 | 119 | 44 | 95 | 235 | 48 | 115 | Asia |
| Janjigian, 2021 | NCT02872116 | 62 (54-69) | 61 (53-68) | 540 | 249 | 560 | 232 | 326 | 462 | 336 | 452 | Asia, USA/Canada, Rest of world |
| Kang, 2022 | NCT02746796 | 64 (25-86) | 65 (27-89) | 253 | 109 | 270 | 92 | 195 | 167 | 194 | 168 | Asia |
| Shitara, 2020 | NCT02494583 | 62 (22-83)^a^;  61 (20-83)^b^ | 62.5 (22-87) | 195^a^;  180^b^ | 62^a^;  76^b^ | 179 | 71 | NA^a^;  NA^b^ | 138^a^;  125^b^ | NA | 135; | Asia, Europe/North America/Australia, Rest of world |
| Sun, 2021 | NCT03189719 | 64 (28-94) | 62 (27-89) | 306 | 67 | 319 | 57 | 149 | 223 | 150 | 225 | Asia, USA |
| Doki, 2022 | NCT03143153 | 64 (40-90) | 64 (26–81) | 253 | 68 | 275 | 49 | 150 | 171 | 154 | 170 | Asia, Rest of world |
| Wang, 2022 | NCT03829969 | 63 (20-75) | 62 (40-74) | 217 | 40 | 220 | 37 | 66 | 191 | 68 | 189 | Asia |
| Luo, 2021 | NCT03691090 | 62 (56-66) | 62 (56-67) | 260 | 38 | 263 | 35 | 71 | 227 | 66 | 232 | Asia |
| Chung, 2022 | NCT03019588 | 61 (32-75) | 61 (37-91) | 32 | 15 | 37 | 10 | 14 | 33 | 12 | 35 | Asia |
| Fuchs, 2022 | NCT02370498 | 62.5 (27-87) | 62.5 (27-87) | 202 | 94 | 208 | 88 | 127 | 169 | 137 | 158 | Asia, Europe/Israel/North America/Australia, Rest of world |
| Bang, 2018 | NCT02625623 | 59 (29-86) | 59 (29-86) | 140 | 45 | 127 | 59 | 66 | 119 | 62 | 124 | Asia, Europe/North America, Rest of world |
| Moehler, 2021 | NCT02625610 | 62 | 61 | 164 | 85 | 167 | 83 | 102 | 147 | 108 | 142 | Asia, Europe/North America, Rest of world |
| Kojima, 2020 | NCT02564263 | 63.0 (23-84) | 62.0 (24-84) | 273 | 41 | 271 | 43 | 126 | 187 | 116 | 197 | Asia, Rest of world |
| Huang, 2020 | NCT03099382 | 60 (54-65) | 60 (54-65) | 20 | 208 | 28 | 192 | 46 | 182 | 44 | 176 | Asia |
| Okada, 2022 | NCT02569242 | 64 (57-69) | 64 (57-69) | 179 | 31 | 185 | 24 | 101 | 109 | 107 | 102 | Asia, Europe/North America |
| Xu, 2022 | NCT03116152 | 60 (54-64) | 60 (54-64) | 88 | 7 | 84 | 11 | 23 | 72 | 23 | 72 | Asia |

NA, no available; ECOG, Eastern Cooperative Oncology Group.

^a^ The therapeutic schedule in experimental group was Pembrolizumab combined with chemotherapy.

^b^ The therapeutic schedule in experimental group was Pembrolizumab monotherapy.
