# Supplementary figures and images for: Efficacy of PD-1/PD-L1 inhibitors in patients with advanced gastroesophageal cancer: An updated meta-analysis based on randomized controlled trials
Source: Front Pharmacol. 2022 Oct 25;13:1009254. doi: 10.3389/fphar.2022.1009254 (PMC9640921; doi:10.3389/fphar.2022.1009254)

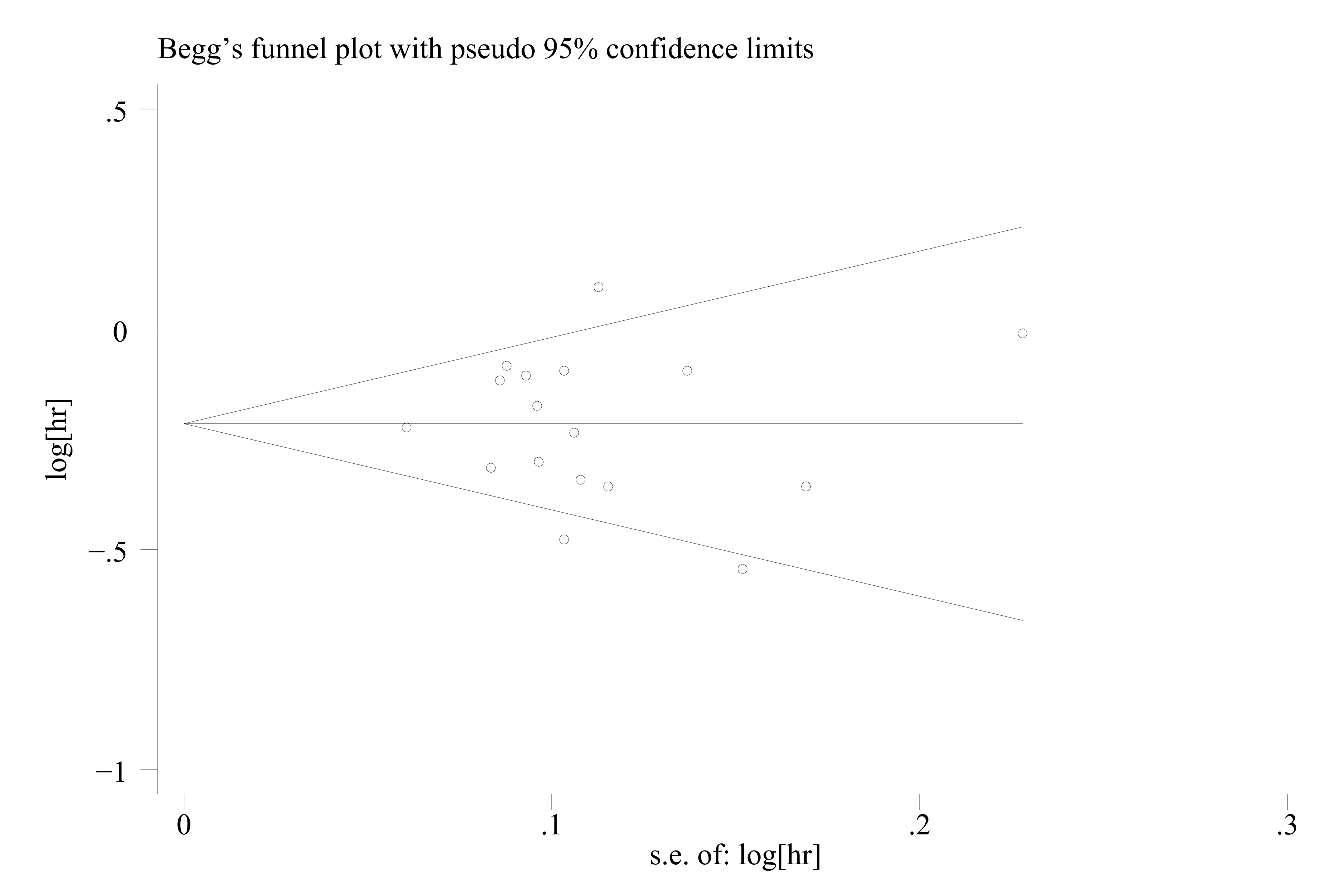

Supplement: Supplementary file 2 [file Image3.TIF]

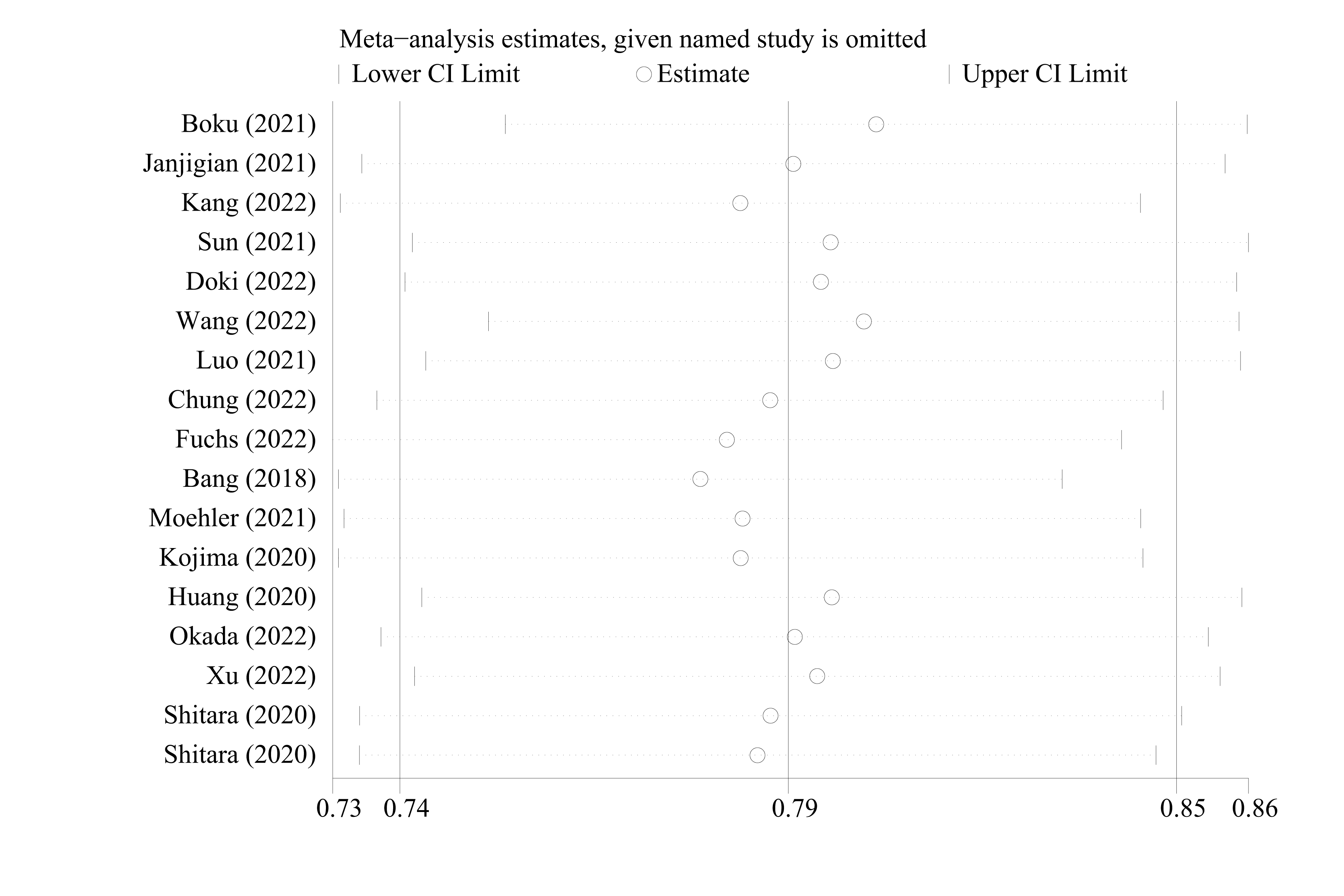

Supplement: Supplementary file 3 [file Image4.TIF]

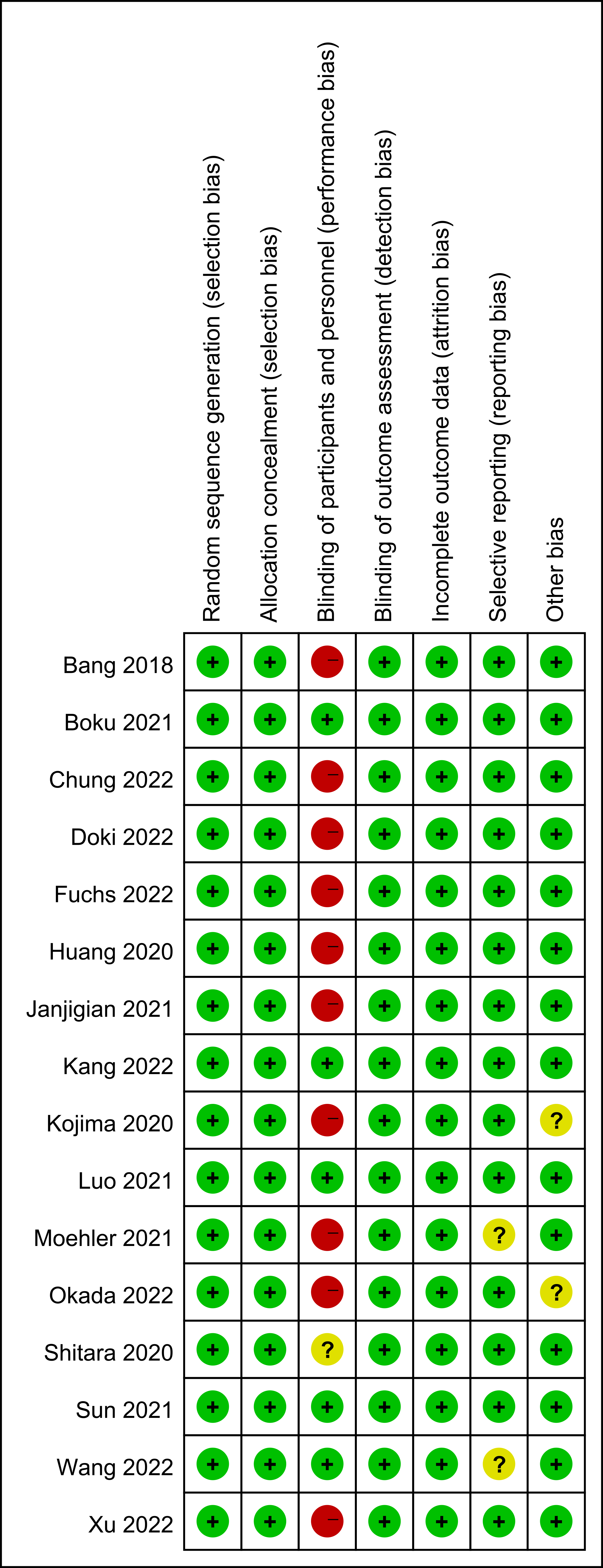

Supplement: Supplementary file 4 [file Image2.TIF]

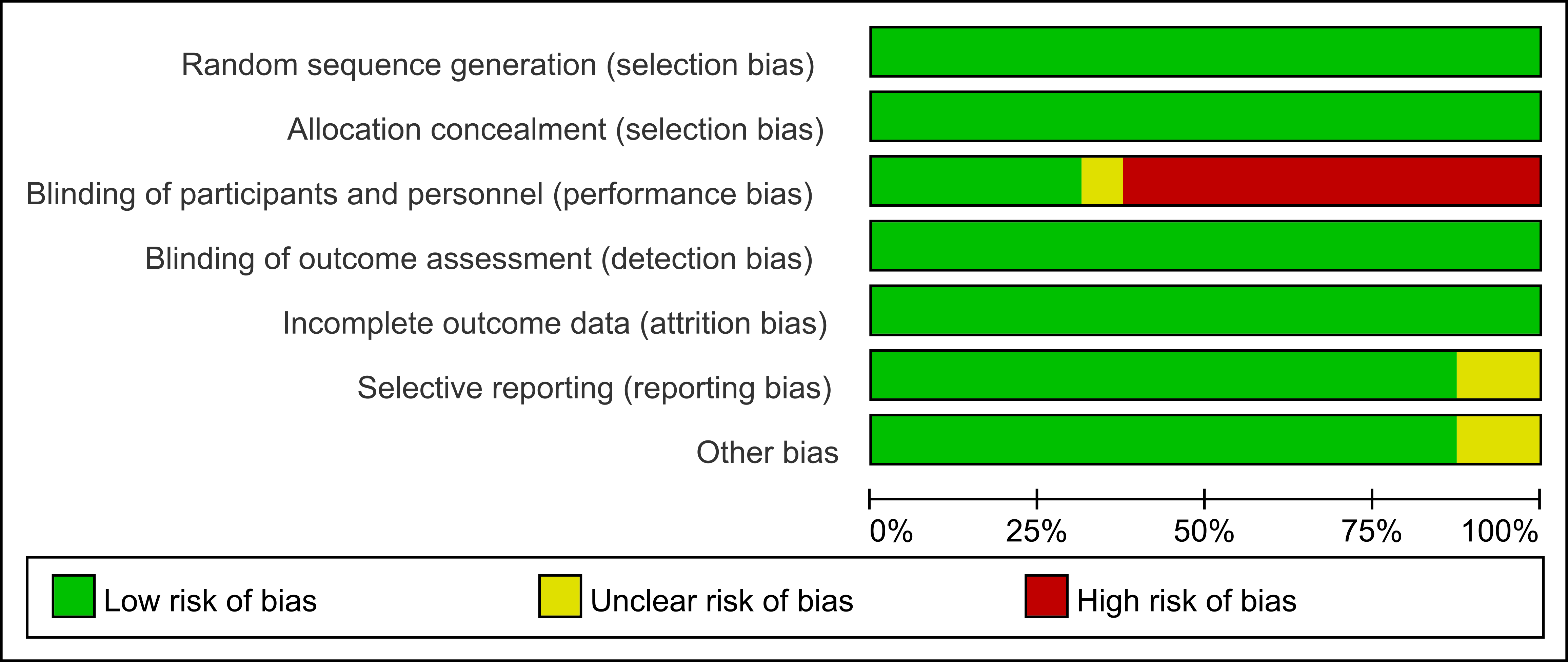

Supplement: Supplementary file 5 [file Image1.TIF]
